# Exploring the Prevalence of Learning Disabilities in a Community Sample of Children Using the Greek Weschler Intelligence Scale for Children (WISC-V GR)

**DOI:** 10.3390/ijerph22030377

**Published:** 2025-03-05

**Authors:** Stavroula Lioliou, Nektaria Pedioti, Kyriaki Vagionaki, Vasiliki Kounali, Nikolaos Bitsakos, Sofia Pitsikaki, Maria Papadakaki

**Affiliations:** Laboratory of Health and Road Safety, Department of Social Work, School of Health Sciences, Hellenic Mediterranean University, 71410 Herakleion, Greece; npedioti@hmu.gr (N.P.); kvagionaki@hmu.gr (K.V.); vkounali@hmu.gr (V.K.); nbitsakos@hmu.gr (N.B.); spitsikaki@hmu.gr (S.P.); mpapadakaki@hmu.gr (M.P.)

**Keywords:** learning disabilities, WISC-V GR, community setting, SLD, ADHD, children, emotional problems, behavioral problems, intelligence

## Abstract

This study aimed to explore the prevalence of learning disabilities (LDs) and the emotional–behavioral difficulties in 208 children from the Crete region in Greece, and who voluntarily presented themselves for study and were evaluated by a university-based interdisciplinary team of mental health professionals. The Greek version of the Wechsler Intelligence Scale for Children–Fifth Edition (WISC-V GR) was used, with its five Primary Index scores and full-scale IQ *(Verbal Comprehension Index, VCI; Visual Spatial Index, VSI; Fluid Reasoning Index, FRI; Working Memory Index, WMI; and Processing Speed, PCI*). Five diagnostic categories were established for the purpose of analysis: *(a) no LDs (TD group), (b) Attention Deficit Hyperactivity Disorder (ADHD), (c) Specific Learning Disabilities (SLDs), (d) Extremely Low FSIQ (below 79), and (e) Emotional/Behavioral difficulties.* The results revealed a 25.5% prevalence of SLDs, 18.75% ADHD, 8.65% Extremely Low FSIQ, and 5.29% emotional/behavioral problems, suggesting that 58% of the study population struggled with some kind of learning difficulty. Statistically significant differences were observed between the “Extremely Low FSIQ” group, the “SLD”, the “ADHD”, and the “TD” diagnostic groups in terms of the “VCI”, “FRI”, and the “FSIQ” scales (*p* < 0.001). Likewise, the “Extremely Low FSIQ” group differed significantly from the “SLD” and “TD” groups in terms of the “VSI”, the WMI, and the “PSI” (*p* < 0.001). The “Behavioural/emotional” and “SLD” groups differed in terms of “VCI” and “Full scale IQ” (*p* < 0.001). The analysis indicated that the children with severe learning difficulties differed from the other groups in terms of their cognitive profiles and that they needed tailor-made educational programs and interventions in a typical classroom. This study offers comparative data from a community sample of children, as well as generated initial evidence from non-clinical settings on the usability and the diagnostic accuracy of the Wechsler Intelligence Scale for Children–Fifth Edition (WISC-V). Further research is suggested. The present study was funded by the Crete Region (MIS 5162111).

## 1. Introduction

Learning disabilities (LDs) are classified within the broader category of neurodevelopmental disorders, with early onset and varying impairments, as well as overall functioning, in the social and cognitive skills of those affected [1]. According to the Diagnostic and Statistical Manual of Mental Disorders, Fifth Edition (DSM-5) [1], the prevalence of LDs is particularly high among school-age children with the Specific Learning Disorder (SLD), affecting 5–15%; and the Attention-Deficit/Hyperactivity Disorder (ADHD), nearly 5% of this population. Both the diagnostic process and the treatment plan remain complex and challenging due to multiple subtypes and definitions, co-occurring conditions, and a lack of standardized criteria and measurements [2,3]. For example, research has indicated that a substantial percentage of individuals with one neurodevelopmental disorder often exhibit symptoms of another, and this overlap poses considerable challenges for healthcare professionals, particularly in terms of accurate differential diagnosis and treatment strategies tailored to address the complex interplay of symptoms [4,5]. A comprehensive epidemiological study on ADHD revealed that 13% of youth also have a comorbid Autism Spectrum Disorder (ASD) diagnosis [6], aligning with the findings of prior research indicating that roughly one in eight young individuals with ADHD also have ASD [7,8,9]. The relationship is even more pronounced in the reverse scenario, with ADHD being the most prevalent comorbidity among children with ASD, occurring in 40–70% of cases [10].

Apart from the complexity deriving from co-occurring conditions, the lack of reliable diagnostic tools and inconsistent diagnostic practices have been assumed to significantly contribute to the misclassification and underdiagnosis of individuals with LDs. In fact, studies indicate that only a fraction of children with learning disabilities are accurately diagnosed and receive the necessary support [11]. Different tests have been found to yield varying results in terms of prevalence, comorbidity, and severity. For example, a study in India found that 10% of children screened positive for SLD, but only 3.08% were positive when more detailed assessment tools were utilized [12]. In other cases, children have been erroneously labeled with emotional issues rather than SLD [13], with such misdiagnoses exacerbating the educational challenges and emotional distress of the children affected. Younger students in a class are sometimes erroneously labeled with learning disabilities when their behavior, in actuality, reflects normal developmental immaturity compared to older classmates [14], while, in other cases, ADHD is classified as a mood disorder, especially in girls [13]. Consequently, research in this domain has been seen as pivotal for developing standardized and comprehensive assessment diagnostic tools that can accurately identify LDs and prevalence estimates and inform appropriate interventions [15].

Children with ADHD often experience significant social challenges, with 50–60% facing peer rejection compared to 13–16% in typical classrooms [16]. This rejection can occur rapidly, sometimes within minutes of initial interactions. Core ADHD symptoms contribute to these difficulties, as children struggle with perspective taking, situational awareness, and appropriate emotional responses. Research shows that children with ADHD are almost twice as likely to have no friends in their classroom. Peer rejection can exacerbate ADHD symptoms over time [17], creating a cycle that may lead to long-term negative outcomes, including academic [18] and emotional problems. Similarly, children with SLDs present with deficits in cognitive skills that interfere with their social life and relations with their peers [19]. Understanding these challenges is crucial for developing effective support strategies to help children with ADHD navigate social situations and improve their long-term outcomes.

Previous studies have reported lower prevalence rates of ADHD and Specific Learning Disabilities in unbiased samples, such as Danielson et al. [20] with 11% for ADHD, Taanila et al. [21] with 8% for ADHD and 19.9% for SLDs, and Gringe et al. [22] with 13.8% for emotional and behavioral problems, providing a comparative framework for interpreting the higher prevalence observed in our study.

What seems most alarming is the emotional burden presented by children with unrecognized LDs [23], who often experience frustration, low self-esteem, anxiety, depression, and academic failure [24,25,26,27]. Research even indicates that the psychological consequences persist into adulthood and can significantly impact quality of life and the general well-being of young adults [25]. Children with comorbid conditions are even at a higher risk of long-term consequences, with a tendency toward experiencing heightened feelings of loneliness, increased victimization, and lower satisfaction with their social interactions [28].

Interestingly, the COVID-19 pandemic has worsened the problems faced by children affected with LDs with the unprecedented disruptions to educational systems and the significant implications for the diagnosis of LDs. Colvin et al. [29] posited that the protracted interruption of conventional educational practices, especially in the Greek context, has potentially rendered traditional diagnostic criteria for LDs inadequate or inapplicable. The ramifications of remote learning modalities on students, particularly those at elevated risk for LDs, remain a subject of ongoing scholarly inquiry. The absence of traditional in-person instructional environments may have obscured LDs that would otherwise be more readily identifiable in conventional educational settings. Despite the resumption of classroom-based instruction, the long-term effects of this disruption to normative teaching experiences on student learning and development remain to be fully elucidated.

In light of the aforementioned challenges, gaining a better understanding of the prevalence, manifestations and trajectories of LDs has been seen as pivotal for improving the diagnosis and treatment of LDs and the overall well-being of affected children [30,31,32,33]. Standardized criteria for assessing co-occurring neurodevelopmental disorders and comprehensive assessments that account for multiple symptoms across different disorders have been warranted in an attempt to reduce the variability in diagnoses across practitioners and to facilitate early intervention [34,35]. This knowledge is also essential for informing policy decisions, allocating resources, and implementing targeted interventions to improve outcomes for individuals with LDs across diverse settings.

Currently, the Wechsler Intelligence Scale for Children–Fifth Edition (WISC-V) [36] provides a comprehensive evaluation framework that is widely employed in both clinical and educational settings, particularly for diagnosing LDs [37]. Despite the fact that LD diagnoses cannot be solely derived from cognitive assessment instruments and intelligence scores, it is anticipated that the WISC-V will further enhance the ability of clinicians and educators to develop targeted interventions and support strategies for children with diverse learning needs. To further enhance its accuracy, refinement and validation is warranted in non-clinical samples. The Greek adaptation of the WISC-V GR was introduced in 2017 [38], providing a culturally relevant instrument for assessing cognitive abilities in Greek children and enabling practitioners to identify potential LDs with enhanced accuracy. However, the implementation and testing of the WISC-V in Greece have, absolutely, been lacking, raising concerns about the adequacy of available evidence for effective diagnosis and intervention.

To the best of our knowledge, this is the first study reporting on the LD prevalence in a community sample of school-aged children in Greece and in Europe generally. Such information is vital for educators, psychologists, and policymakers to further improve diagnostic procedures, but also to develop targeted interventions and support systems that address the specific needs of children with LDs. The implementation of early screening strategies in community settings for the general population remains critically important to mitigate the additional psychological burden associated with severe learning disabilities and late diagnosis. The present study is a part of a 4-year project called “Personalized psychosocial support and counseling for learning disabilities”, which is being funded by the Crete Region [39,40,41]. The primary objective of this study is to apply the WISC-V GR to explore the prevalence of LDs in a community sample of children. Emphasis is placed on ADHD and SLDs due to their high prevalence among school-aged children, as well as on the emotional and behavioral difficulties recorded as the primary diagnosis among this population [1]. By examining initial data from non-clinical settings in Greece, this research seeks to provide insights into the cognitive profiles of Greek children and to the prevalence of LDs within this population. The current research will further offer useful insights on WISC-V GR application in a community setting.

## 2. Materials and Methods

### 2.1. Procedures

This study employed a cross-sectional design to examine the prevalence of learning disabilities in Greece using the WISC-V GR, and it was funded by the Crete Region (MIS 5162111). It is important to note that the participants in this study were recruited from families who had voluntarily sought evaluation for learning disabilities through a community-based service. This recruitment method may introduce selection bias, as families concerned about their child’s development are more likely to seek such services. Consequently, the prevalence rates observed in this study may reflect an over-representation of learning difficulties compared to an unbiased population. The study participants were selected from a group that utilizes a unique community-based mobile assessment service: the interdisciplinary Mobile Unit of the Hellenic Mediterranean University in the Crete Region of Greece. This service, through local municipal Social Services Departments, evaluates children for learning disabilities at no cost. The multidisciplinary team comprised school psychologists, social workers, special education teachers, and speech therapists, who conducted assessments by appointment across all of the municipalities in Crete from 2020 till 2023. Families could voluntarily request an evaluation for their children. It is the only unit of its kind in Greece.

Upon obtaining written parental consent, the team conducted educational and psychological assessments of the children. More specifically, all of the assessments were conducted in controlled environments within educational institutions or municipal facilities, as provided by local community services. Access to these venues was authorized by the local administration office, ensuring standardized testing conditions. Each examiner was allocated a private room to conduct tests and interviews, maintaining consistency and confidentiality across all sessions.

The study’s inclusion criteria were as follows: Eligible participants were children between the ages of 4 and 12 years who had not undergone an evaluation for learning disabilities within the past two years. Consequently, assessments conducted prior to this two-year window were not taken into account. This approach ensured that all participants were evaluated based on current data, regardless of any historical assessments that may have existed. In that sense, all of the children were treated as “new entries”. Additionally, children were required to be residents of the Crete region and fluent in Greek. Finally, both the availability and willingness of the children and their families to participate in this study were essential for inclusion.

### 2.2. Research Instruments

For the purposes of this study, the Wechsler Intelligence Scale for Children (WISC-V GR) [38] was administered by a trained school psychologist to assess cognitive abilities. The test was delivered in a one-on-one setting, adhering to standardized protocols in a quiet room with appropriate furnishings and adequate lighting, and it lasted approximately 50–60 min. WISC-V comprises the following 5 Primary Index scores and a Full-Scale IQ as key components designed to evaluate the various cognitive abilities in children: (a) the Verbal Comprehension Index (VCI), including the Similarities and Vocabulary subtests, which assesses a child’s verbal reasoning capabilities; (b) the Visual Spatial Index (VSI), formed by the Block Design and Visual Puzzles subtests, which measures a child’s visuospatial reasoning skills; (c) the Fluid Reasoning Index (FRI), consisting of the Matrix Reasoning and Figure Weights subtests, which provides an assessment of inductive and quantitative reasoning abilities; (d) the Working Memory Index (WMI), incorporating the Digit Span and Picture Span subtests, which evaluates the child’s working memory capacity; and (e) the Processing Speed Index (PSI), comprising the Coding and Symbol Search subtests, which assesses both cognitive processing speed and motor speed. WISC has long been a foundational tool in assessing children’s cognitive abilities [42,43]. Its most recent edition, the WISC-V [36], provides a comprehensive evaluation framework that is widely employed in both clinical and educational settings, particularly for diagnosing learning disabilities [37].

The educational assessment followed similar guidelines and conditions, and they were conducted by a special education teacher in a one-on-one setting, with each session lasting approximately 75 min. The child’s reading, writing, and mathematical skills were evaluated using standardized tools. The selection of “Test Alpha” (reading test) [44] and the “Psychometric test of mathematical competence for children and adolescents” [45] was guided by the need for reliable, valid, and comprehensive assessment tools that provide detailed insights into students’ reading and mathematical abilities. Both tools have established psychometric properties, are user-friendly, and support the development of targeted instructional interventions. “Test Alpha” evaluates word decoding, fluency, morphology and syntax, and reading comprehension, whereas the math test assesses lexical knowledge, calculations, and problem solving.

Similarly, a social worker conducted interviews with the parents, adhering to the same environmental standards, with these sessions lasting approximately 90 min. The Child Behavior Checklist (CBCL) [46] was also administered. CBCL is a widely used assessment tool designed to identify behavioral and emotional problems in children and adolescents. It is part of the Achenbach System of Empirically Based Assessment (ASEBA). For the purpose of this study, the school-age children version (aged 6 to 18 years) for parents was filled out with help of a social worker. It consists of 113 questions scored on a three-point Likert scale, typically taking 10–20 min to complete. The CBCL/6–18 includes eight syndrome scales grouped into internalizing and externalizing problems, as well as DSM-oriented scales and competence scales. The 2001 revision added multicultural norms to allow for cross-cultural comparisons. The CBCL has demonstrated high reliability and validity across many cultural contexts, and it is widely used in clinical practice, research, and as a diagnostic screener. The overall methodological approach ensured consistency, privacy, and optimal conditions for all the participants across the various assessment components.

The diagnostic classification of children into distinct groups was informed by a comprehensive evaluation process. This process integrated data from multiple assessments and clinical interviews. The final determination of each child’s diagnostic category was made during an interdisciplinary team meeting, which convened after the completion of all assessment procedures. This collaborative approach ensured that all relevant information (test results, clinical observations, and developmental history) was considered in the diagnostic decision-making process.

### 2.3. Statistical Analysis

The statistical analysis was performed utilizing IBM SPSS, version 24, for Windows. One-way ANOVA and Tukey’s HSD test for multiple comparisons were conducted. Five diagnostic categories were established: (a) the Typically Developing (TD) group, consisting of children without significant difficulties; (b) the ADHD group, including children whose primary diagnosis was ADHD; (c) the SLD group, comprising children with Specific Learning Disabilities; (d) the Extremely Low IQ group, including children with an IQ below 79; and (e) the Emotional/Behavioral group, encompassing children primarily diagnosed with emotional or behavioral challenges.

Cases with incomplete WISC-V GR assessments were excluded from the statistical analyses requiring full datasets. However, the available data from partially completed assessments were included in descriptive summaries where appropriate. No imputation techniques were used to estimate the missing values [47].

One-way ANOVA was conducted to compare the performance of the groups in terms of the Primary Index scores, Full-Scale IQ scores, and Subtest scores in WISC-V GR. The Tukey’s HSD test for multiple comparisons was used for post hoc analysis to determine specific group differences in the Primary Index scores, Subtest scores, and Full-Scale IQ scores.

## 3. Results

A total of 208 children, all fluent in Greek, participated in the assessment process (mean age = 9.23 years; SD = 1.565). Some children were unable to complete the WISC-V GR assessment due to technical difficulties, medical issues, time constraints, or other unforeseen circumstances.

The assessment process yielded the following diagnostic outcomes: 76 children exhibited no or minor learning or developmental difficulties and were categorized as the “Referral” group. A total of 39 children were diagnosed with ADHD, forming the “ADHD” group. Next, 53 children were identified with Specific Learning Disabilities (SLDs), comprising the “SLD” group. A total of 18 children had an IQ below 79, and they were classified in the “Extremely Low FSIQ” group, while 11 children with primary diagnoses of emotional or behavioral difficulties were assigned to the “Emotional/Behavioral” group. Of the participating children, 63.5% were male and 36.5% were female. Additionally, 187 parents participated in this study (mean age = 41.9 years; SD = 5.3), with a gender distribution of 7.0% male and 93.0% female. In terms of parental occupations, the most common sectors were public sector employment (29.0%) and private sector employment (31.8%). The majority of children resided in rural or semi-urban areas (65.8%), while a smaller proportion lived in urban areas (34.2%). Regarding educational attainment, parents exhibited diverse backgrounds: 3.9% had completed elementary education, 33.5% had a high school diploma, and 62.6% had attained a university-level education. Family structure data revealed that most families were married (83.4%), while smaller proportions were divorced (13.8%), single (1.1%), or in other family arrangements (1.1%). Financial satisfaction among parents varied: 8.7% reported being extremely satisfied with their financial situation, while 6.8% reported being extremely dissatisfied. These demographic characteristics provide an overview of the children and their families included in the dataset, as summarized in Table 1.

Out of the 208 children assessed, some were unable to complete all WISC-V GR subtests due to various factors, such as technical difficulties or time constraints. These cases were excluded from the respective analyses but were considered in overall descriptive statistics where applicable. The final sample size for each analysis is indicated in Table 2.

Given the potential for sample bias, we compared our findings to those reported in the literature on unbiased samples of children, both in Greek and international contexts. Studies have reported lower distributions of SLDs [21], ADHD [20,21], and emotional/behavioral difficulties [22]. suggesting that, while the overall prevalence in our sample was higher, the relative incidence of each type of difficulty aligned with the broader research.

A one-way ANOVA was conducted to compare the performance of the groups in terms of the Primary Index scores, Full-Scale IQ scores, and Subtest scores in the WISC-V GR. The ANOVA revealed a statistically significant difference in the mean score between at least two groups in the Verbal Comprehension Index F(4,186) = 7.24, *p* < 0.001; the Visual Spatial Index F = 4.69, *p* = 0.001; the Fluid Reasoning Index F = 5.79, *p* < 0.009; and the Full-Scale IQ F = 8.97, *p* < 0.001. Significant differences were also found in the five subtests: Similarities F(4,185) = 6.41, *p* < 0.001; Digit Span F = 3.85, *p* = 0.005; Visual Puzzles F(4,184) = 5.26, *p* < 0.001; Matrix Reasoning F = 4.51, *p* = 0.002; and Vocabulary F = 4.85, *p* = 0.001.

Tukey’s HSD test for multiple comparisons found that the mean value of the Verbal Comprehension Index (VCI) was significantly statistically different between the Extremely Low FSIQ group (M = 84.65, SD = 12.72), the SLD group (M = 101.84, SD = 12.6), the ADHD group (M = 95.03, SD = 11.8), and the Referral group (M = 99.43, SD = 12.6), with *p* < 0.001, F = 7.24. There was also a significant statistical difference between the SLD group and the Emotional/Behavioral group (M = 91, SD = 17.26). In the Visual Spatial Index (VSI), the Extremely Low FSIQ group (M = 86.18, SD = 10.32) differed statistically significantly from the SLD group (M = 103.04, SD = 13.39) and the TD group (M = 98.28, SD = 16.82), with *p* = 0.001, F = 4.68. In the Fluid Reasoning Index (FRI), the Extremely Low FSIQ group (M = 81.29, SD = 15.43) was significantly statistically different from the SLD (M = 98.60, SD = 14.53), ADHD (M = 93.85, SD = 14.78), and TD groups (M = 96.48, SD = 13.39), with F = 5.58, *p* < 0.001. In the Working Memory Index (WMI), the Extremely Low FSIQ group (M = 75.94, SD = 14.06) was significantly statistically different from the SLD group (M = 86.14, SD = 13.07) and the TD group (M= 87.47, SD = 12.42). In terms of the Processing Speed Index (PSI), the Extremely Low FSIQ group (M = 82, SD = 14.19) was significantly statistically different from the SLD group (M = 94.74, SD = 10.26) and the TD group (M = 94.43, SD = 13.37).

Significant statistical differences were also observed in the Full-Scale IQ score, found in Table 3, statistically signifcant differences in the Similarities (F = 8.97, *p* < 0.001) between the Extremely Low FSIQ group (M = 78.41, SD = 14.33), the SLD group (M = 96.36, SD = 11.46), the Referral group (M = 95.08, SD = 11.39), and the ADHD group (M = 90.44, SD = 12.49). There was also a significant difference between the SLD group and the Emotional/Behavioral group (M = 85.40, SD = 15.49).

Tukey’s HSD test was used for multiple comparisons in the Subtest scores, found in Table 4, statistically significant differences in the Similarities (F = 6.41, *p* < 0.001) between the Extremely Low FSIQ group (M = 6.94, SD = 2.99), the SLD group (M = 10.57, SD = 2.85), and the Referral group (M = 10.35, SD = 3.24) were found. In the Digit Span subtest (F = 3.84, *p* = 0.005), there was a difference between the Extremely Low FSIQ group (M = 5.44, SD = 3.36), the SLD group (M = 7.63, SD = 2.30), and the Referral group (M = 7.93, SD = 2.45). In the Visual Puzzles subtest (F = 5.26, *p* < 0.001), there were statistically significant differences between the Extremely Low FSIQ group (M = 7.06, SD = 2.72), the SLD group (M = 10.73, SD = 2.85), and the Referral group (M = 9.88, SD = 3.16). A similar pattern was followed in the Matrix Reasoning subtest (F = 4.51, *p* = 0.002). More specifically, statistically significant differences were observed between the Extremely Low FSIQ group (M = 6.44, SD = 3.54), the SLD group (M = 9.73, SD = 3.01), and Referral group (M = 9.27, SD = 2.74). In the Vocabulary subtest (F = 4.85, *p* = 0.001), the following differences between the groups were observed: the Extremely Low FSIQ group with M = 7.25 and SD = 2.59; the SLD group with M = 9.75 and SD = 2.13; and the Referral group with M = 9.39 and SD = 2.07.

## 4. Discussion

Our study reveals several significant findings regarding the prevalence and identification of LDs in the Greek educational context. Strikingly, 58% of the students assessed were identified with some kind of learning difficulty, a finding that demands careful interpretation given the context of the COVID-19 pandemic and its disruptions to Greek education. This result underscores the critical need for comprehensive learning disability screening protocols and educational policy reforms.

Firstly, this study indicates that approximately 25% of the sampled population exhibited some form of SLD, a rate that is substantially higher than international estimates [1]. This discrepancy warrants further examination and may be elucidated by the research of Anastasiou and Polychronopoulou [48], which suggests a disproportionate prevalence of SLD in reading and writing among Greek secondary school students compared to their elementary counterparts.

A closer look at the cognitive profiles of the five diagnostic categories revealed clear differences across tasks. The TD group showed stronger performance in verbal comprehension, working memory, and processing speed compared to the SLD group. Table 2 shows that, while the SLD group had relatively higher scores in the visual, spatial, and fluid reasoning tasks, their lower scores in digit span (working memory) and coding (processing speed) reflected the challenges they face in retaining and processing information quickly. In contrast, the TD group’s more balanced profile across all indices highlighted the cognitive gaps seen in the children with SLD, especially in tasks requiring memory retention and rapid information processing. This distinction helps explain the academic struggles often observed in children with SLD, even when they demonstrate strengths in certain reasoning tasks.

Several factors may contribute to the observed discrepancies in SLD prevalence, including the lack of standardized evaluation procedures, the insufficient training of professionals in SLD identification, the influence of cultural and educational factors in diagnosis [49], and the self-selection bias of the sample. These findings underscore the imperative for a comprehensive, standardized approach to SLD identification and intervention across all educational levels.

The Centers for Interdisciplinary Assessment, Counseling and Support (KE.DA.SY.) play a crucial role in the diagnosis of learning disabilities in Greece. Established in 2018 and subsequently reorganized under Law 4823/2021 (Article 11, Section 1-1—https://search.et.gr/el/fek/?fekId=603011, accessed on 24 February 2025), these centers are responsible for the timely diagnosis of learning disabilities in children upon referral by their teachers, albeit with parental consent. However, the current operational framework of KE.DA.SY. presents significant challenges in the early identification and intervention for students with SLD. These centers face substantial limitations in both financial and human resources, impeding the implementation of universal screening protocols across the student population. In fact, the primary responsibility for identifying students at high risk for learning disabilities falls predominantly on classroom teachers, potentially resulting in a considerable number of students remaining undiagnosed. Consequently, some students may only be referred for assessment upon entering secondary education, where their academic impairments become more pronounced and thus easier to detect [48]. The systemic limitations in early identification have profound implications for student prognoses and academic trajectory. A prolonged period without appropriate intervention often leaves affected individuals with coexisting psychological consequences, including persistent feelings of frustration, disappointment, and diminished self-esteem [24]. These findings highlight the critical need for improved early identification and intervention strategies for students with LDs in the Greek educational system.

Recent research in Greece has shifted focus toward examining teacher knowledge gaps regarding Attention Deficit Hyperactivity Disorder (ADHD). The same findings are being observed in studies at university level [50]. While some studies have reported improvements in teachers’ understanding of ADHD, overall knowledge levels remain moderate, potentially leading to under-referrals of children with the disorder. Giannopoulou et al. [51] observed enhanced teacher knowledge compared to earlier investigations by Kakouros et al. [52,53]. However, this improvement may not be sufficient to ensure accurate identification and referral of students with ADHD.

Interestingly, another finding of this study identified a relationship between emotional/behavioral challenges and cognitive functioning. Research on this matter is still scarce. Individuals in the Emotional/Behavioral group in our study demonstrated specific cognitive vulnerabilities, particularly in verbal comprehension and overall intellectual functioning. These cognitive impairments may be associated with their emotional or behavioral difficulties [54]. This finding emphasizes the importance of distinguishing between cognitive profiles in various neurodevelopmental and emotional–behavioral conditions, as it may have implications for assessment, diagnosis, and intervention strategies [23].

Additionally, no statistically significant disparities were found in the Full-Scale Intelligence Quotient (FSIQ) scores among the SLD, the ADHD, and the TD groups. This finding aligns with both historical research [55] and recent studies utilizing WISC-V [56]. However, it stands in contrast to a 2020 Greek study [57] that employed the WISC-III GR, which reported statistically significant differences between children with ADHD and both TD children and those with SLD in writing or reading across the majority of subtests.

The inconsistencies observed across these studies potentially underscore the intricacy of cognitive profiles in neurodevelopmental disorders. They also highlight the potential influence of assessment tools, cultural contexts, and sample characteristics on research outcomes. A more comprehensive statistical analysis could have yielded different results. For instance, a study examining the WISC-IV cognitive profiles of children with SLD compared to TD children suggested that merely comparing FSIQ scores may be insufficient for detecting cognitive discrepancies between groups [58]. The researchers proposed that analysis of the General Ability Index (GAI) was additionally necessary to inform diagnosis.

These findings collectively emphasize the importance of employing multiple measures and indices when assessing cognitive profiles in neurodevelopmental disorders. They also suggest that reliance on a single composite score, such as FSIQ, may not fully capture the nuanced cognitive differences that exist between these groups. The cognitive differences highlighted between the TD and SLD groups emphasize the need for tailored interventions that address specific areas such as working memory and processing speed, which are often weaker in children with SLD. Future research in this area may benefit from incorporating more comprehensive assessment strategies and considering a wider range of cognitive indices to better elucidate the complex cognitive profiles associated with SLD and ADHD.

### Limitations and Strengths of the Study

This study has several limitations that should be considered when interpreting the results. The primary limitation is the small sample size, which may have reduced the statistical power of the study to detect significant effects potentially limiting the generalizability of the findings. Additionally, this study was conducted in a single region, which may differ from other country regions, thus potentially influencing the generalizability of results. The regional focus may have introduced bias due to the specific cultural, socioeconomic, or environmental factors unique to the area. Furthermore, a significant source of potential bias stems from the nature of the sample itself. Families who participated in the program were likely predisposed to consider the possibility of learning disabilities as they voluntarily sought out the assessment services. This self-selection bias may have led to an over-representation of children with learning difficulties in the sample, potentially inflating the observed prevalence rates. These limitations suggest that the findings should be interpreted cautiously and may not be representative of broader populations or contexts. Nevertheless, though the self-selection bias inherent in our sample may have led to an over-representation of children with learning difficulties, our comparison with studies conducted on unbiased populations provides a more nuanced understanding. This comparison indicates that although our sample may include more severe cases, the relative incidence of each learning difficulty is consistent with broader research.

Another limitation of this study is the presence of missing data, as some children were unable to complete the full WISC-V GR assessment. These cases were excluded from the statistical analyses that required complete datasets, which may have introduced some bias. It is possible that children who did not complete the assessment had different cognitive profiles or more severe difficulties that affected their ability to participate fully. While this is unlikely to have significantly impacted the overall patterns observed, it may have influenced certain comparisons.

This study marks the first community-based mapping of the prevalence of learning disabilities in Greece using the latest Greek version of the Wechsler Intelligence Scale for Children, Fifth Edition (WISC-V GR). Its novelty is multifaceted: it represents an initial attempt to establish learning disability prevalence rates within a community sample; it employed current, standardized, and accurate diagnostic tools that are often absent in public services; and it adopted an innovative service delivery model. By bringing assessments directly to families, this study overcame geographical barriers for those in remote areas and provided access to economically disadvantaged families who might otherwise have been unable to afford such evaluations. This approach not only produced valuable data, but it also addressed critical issues of accessibility and equity in the assessment of learning disabilities.

## 5. Conclusions

This research contributes to the growing body of evidence supporting more comprehensive diagnostic approaches in learning disabilities, particularly in the context of educational disruptions. It offers valuable data on the applicability of the WISC-V GR in diverse settings and helps bridge the gap between clinical and non-clinical research in understanding children’s learning difficulties. Importantly, the current study generates evidence on the importance of implementing community-based studies using standardized instruments like the WISC-V GR for establishing accurate prevalence rates, identifying at-risk individuals, and developing targeted interventions for learning disabilities. Community studies offer a proactive approach to early detection, contrasting with the traditional reactive model of waiting for referrals. This method allows for the screening of LDs among the general population of students, potentially uncovering difficulties in cognitive functions, academic skills, and neurodevelopmental disorders at earlier stages. This study concludes in suggesting that fundamental policy alterations are needed. Future research should aim to replicate these results with larger, more diverse samples across multiple regions, as well as employ strategies to mitigate self-selection bias to enhance the robustness and generalizability of the findings.

## Figures and Tables

**Table 1:** The sociodemographic characteristics of the participants.

**Children**	
Age	M = 9.23 (SD = 1.5, min/max 4–12)
Gender (Male)	132 (63.5%)
**Residence**	
Rural/semi-urban	123 (65.8%)
Urban	64 (34.2%)
**Parents’ Family Status**	
Married	151 (83.4%)
Divorced	25 (13.8%)
Single	2 (1.1%)
Other	2 (1.1%)
Widow/er	1 (0.6%)
Missing	6
**Parents**	
Age	M = 41.9 (SD = 5.3, min = 29, max = 59)
Gender (Female)	174 (93.0%)
**Diagnosis**	
ADHD	39 (18.75%)
SLD	53 (25.5%)
Extremely Low FSIQ	18 (8.65%)
Emotional/Behavioral	11 (5.29%)
Typically Developing (TD)	76 (36.5%)
Missing	11 (5.29%)

**Table 2:** The means, standard deviations, and one-way analyses of variance in the WISC-V GR (Primary Index Scores, FSIQ, and Subtests).

	TD	ADHD	SLD	Extremely Low FSIQ	Behavioral/Emotional		
**N**	75	39	50	17	10		
	M (SD)					F	*p*
**Index Scores**							
VCI	99.43 (12.6)	95.03 (11.8)	101.84 (12.6)	84.65 (12.72)	91 (17.26)	7.241	<0.001
VSI	98.28 (16.82)	95.18 (12.18)	103.04 (13.39)	86.18 (10.32)	97 (15.74)	4.685	0.001
FRI	96.48 (13.39)	93.85 (14.78)	98.60 (14.53)	81.29 (15.43)	88 (15.03)	5.79	<0.001
WMI	87.47 (12.42)	84.56 (10.76)	86.14 (13.07)	75.94 (14.06)	80.10 (12.92)	3.481	0.009
PSI	94.43 (13.37)	92.62 (16.49)	94.74 (10.26)	82 (14.19)	86.60 (14.40)	3.625	0.007
FSIQ	95.08 (11.39)	90.44 (12.49)	96.36 (11.46)	78.41 (14.33)	85.40 (15.49)	8.969	<0.001
**N**	74	39	51	16	10		
	M (SD)					F	*p*
**Subtests**							
Block Design	10.05 (2.33)	9.13 (2.52)	10.31 (2.54)	8.00 (1.96)	9.50 (2.79)	3.708	0.006
Similarities	10.35 (3.24)	8.77 (2.86)	10.57 (2.85)	6.94 (2.99)	8.40 (3.77)	6.414	<0.001
Digit Span	7.93 (2.45)	7.15 (2.31)	7.63 (2.30)	5.44 (3.36)	6.60 (2.36)	3.847	0.005
Coding	8.86 (3.19)	8.38 (3.36)	8.86 (2.45)	6.69 (3.61)	7.00 (3.19)	2.318	0.050
Figure Weights	9.59 (2.64)	9.03 (3.00)	9.86 (2.72)	7.19 (2.56)	8.20 (2.44)	3.652	0.007
Symbol Search	9.25 (2.88)	8.67 (3.12)	9.22 (2.31)	6.60 (2.87)	8.30 (2.86)	3.157	0.015
Picture Span	7.82 (2.69)	7.36 (2.40)	7.63 (2.92)	6.25 (2.40)	6.50 (2.95)	1.516	0.199
Visual Puzzles	9.88 (3.16)	9.08 (2.70)	10.73 (2.85)	7.06 (2.72)	9.40 (3.06)	5.259	<0.001
Matrix Reasoning	9.27 (2.74)	8.82 (2.79)	9.73 (3.01)	6.44 (3.54)	7.70 (3.36)	4.513	0.002
Vocabulary	9.39 (2.07)	9.38 (2.46)	9.75 (2.13)	7.25 (2.59)	7.80 (2.86)	4.852	0.001

There was homogeneity of variances between the groups, as assessed by Levene’s test for equality of the variances (*p* > 0.05).

**Table 3:** The post hoc Tukey’s test results for the Primary Index scores and Full-Scale IQ scores in WISC-V GR.

N	GROUPS	VCI	VSI	FRI	WMI	PSI	FSIQ
75	TD	bc	b	b	b	b	bc
39	ADHD	bc	ab	b	ab	ab	bc
50	SLD	c	b	b	b	b	c
17	Extremely Low FSIQ	a	a	a	a	a	a
10	Behavioral/Emotional	ab	ab	ab	ab	ab	ab
	F	7.24	4.68	5.58	3.49	3.62	8.97
	*p*	<0.001	0.001	<0.001	0.009	0.007	<0.001

Different letters show the differences between the corresponding groups of children according to the post hoc Tukey’s test, with groups that share the same letter having not differed significantly, while groups with different letters exhibited statistically significant differences.

**Table 4:** The post hoc Tukey’s test results for the Subtest scores in WISC-V GR.

	TD	ADHD	SLD	Extremely Low FSIQ	Behavioral/ Emotional		
**Subtests**	**75**	**39**	**50**	**16**	**10**	**F**	***p***
Block Design	b	ab	b	a	ab	3.70	0.006
Similarities	b	ab	b	a	ab	6.41	<0.001
Digit Span	b	ab	b	a	ab	3.84	0.005
Coding	a	a	a	a	a	2.41	0.050
Figure Weights	b	ab	b	a	ab	3.65	0.007
Symbol Search	b	ab	b	a	ab	3.15	0.015
Picture Span	a	a	a	a	a	1.51	0.199
Visual Puzzles	b	ab	b	a	ab	5.26	<0.001
Matrix Reasoning	b	ab	b	a	ab	4.51	0.002
Vocabulary	bc	bc	c	a	ab	4.85	0.001

Different letters show the differences between the corresponding groups of children according to the post hoc Tukey’s test, with groups that share the same letter having not differed significantly, while groups with different letters exhibited statistically significant differences.

## Data Availability

The data that support the findings of this study are available from the corresponding authors upon reasonable request.

## Abbreviations

The following abbreviations are used in this manuscript:

LD	Learning Disability
SLD	Specific Learning Disorder
ADHD	Attention Deficit Hyperactivity Disorder
TD	Typically Developing
FSIQ	Full-Scale Intelligence Quotient
VCI	Verbal Comprehension Index
FRI	Fluid Reasoning Index
PSI	Processing Speed Index
WMI	Working Memory Index
VSI	Visual Spatial Index
